# Incidence of immune-mediated inflammatory diseases following COVID-19: a matched cohort study in UK primary care

**DOI:** 10.1186/s12916-023-03049-5

**Published:** 2023-09-21

**Authors:** Umer Syed, Anuradhaa Subramanian, David C. Wraith, Janet M. Lord, Kirsty McGee, Krishna Ghokale, Krishnarajah Nirantharakumar, Shamil Haroon

**Affiliations:** 1 Institute of Applied Health Research, College of Medical and Dental Sciences, University of Birmingham, Edgbaston, Birmingham, B15 2TT UK; 2 Institute of Immunology and Immunotherapy, College of Medical and Dental Sciences, University of Birmingham, Birmingham, UK; 3 NIHR Birmingham Biomedical Research Centre, University Hospital Birmingham and University of Birmingham, Birmingham, UK; 4 MRC-Versus Arthritis Centre for Musculoskeletal Ageing Research, Institute of Inflammation and Ageing, University of Birmingham, Birmingham, UK

**Keywords:** COVID-19, SARS-CoV-2, Cohort study, Immune-mediated inflammatory diseases

## Abstract

**Background:**

Some patients infected with severe acute respiratory syndrome coronavirus-2 (SARS-CoV-2) go on to experience post-COVID-19 condition or long COVID. Preliminary findings have given rise to the theory that long COVID may be due in part to a deranged immune response. In this study, we assess whether there is an association between SARS-CoV-2 infection and the incidence of immune-mediated inflammatory diseases (IMIDs).

**Methods:**

Matched cohort study using primary care electronic health record data from the Clinical Practice Research Datalink Aurum database. The exposed cohort included 458,147 adults aged 18 years and older with a confirmed SARS-CoV-2 infection and no prior diagnosis of IMIDs. They were matched on age, sex, and general practice to 1,818,929 adults with no diagnosis of confirmed or suspected SARS-CoV-2 infection. The primary outcome was a composite of any of the following IMIDs: autoimmune thyroiditis, coeliac disease, inflammatory bowel disease (IBD), myasthenia gravis, pernicious anaemia, psoriasis, rheumatoid arthritis (RA), Sjogren’s syndrome, systemic lupus erythematosus (SLE), type 1 diabetes mellitus (T1DM), and vitiligo. The secondary outcomes were each of these conditions separately. Cox proportional hazard models were used to estimate adjusted hazard ratios (aHR) and 95% confidence intervals (CI) for the primary and secondary outcomes, adjusting for age, sex, ethnic group, smoking status, body mass index, relevant infections, and medications.

**Results:**

Six hundred and nighty six (0.15%) and 2230 (0.12%) patients in the exposed and unexposed cohort developed an IMID during the follow-up period over 0.29 person-years, giving a crude incidence rate of 4.59 and 3.65 per 1000 person-years, respectively. Patients in the exposed cohort had a 22% increased risk of developing an IMID, compared to the unexposed cohort (aHR 1.22, 95% CI 1.12 to 1.33). The incidence of three IMIDs was significantly associated with SARS-CoV-2 infection. These were T1DM (aHR 1.56, 1.09 to 2.23), IBD (aHR 1.36, 1.18 to 1.56), and psoriasis (1.23, 1.05 to 1.42).

**Conclusions:**

SARS-CoV-2 was associated with an increased incidence of IMIDs including T1DM, IBD and psoriasis. However, these findings could be potentially due to ascertainment bias. Further research is needed to replicate these findings in other populations and to measure autoantibody profiles in cohorts of individuals with COVID-19.

**Supplementary Information:**

The online version contains supplementary material available at 10.1186/s12916-023-03049-5.

## Summary box

What is already known on this topic

- A subsection of the population infected with SARS-CoV-2 go on to experience post-COVID-19 condition or long COVID.
- Preliminary findings, such as case reports of post-COVID-19 immune-mediated inflammatory diseases, increased autoantibodies in COVID-19 patients, and molecular mimicry of the SARS-CoV-2 virus have given rise to the theory that long COVID may be due in part to a deranged immune response.

What this study adds

- SARS-CoV-2 infection was associated with a 22% relative increase in the risk of developing certain immune-mediated inflammatory diseases, including type 1 diabetes mellitus, inflammatory bowel disease, and psoriasis.
- These findings support the hypothesis that a subgroup of long COVID may be caused by immune-mediated inflammatory mechanisms.

## Background

Emerging in late 2019, severe acute respiratory syndrome coronavirus-2 (SARS-CoV-2), the virus causing the coronavirus disease-2019 (COVID-19) pandemic, as of March 2023, resulted in over 6 million deaths worldwide [1, 2]. The acute presentation can range from being completely asymptomatic to sepsis, organ failure and death [3]. The effects of COVID-19 are not limited solely to acute infection but have also manifested in a series of post-acute sequelae commonly referred to as long COVID or post-COVID-19 condition [4, 5].

The World Health Organisation define this as symptoms occurring in people with a history of probable or confirmed SARS-CoV-2 infection three months after the onset of COVID-19 that cannot be explained by an alternative diagnosis [5, 6]. With over a third of people with COVID-19 reporting persistent symptoms and over 1.7 million UK residents self-reporting the condition, long COVID is emerging as one of the major public health challenges of the modern era [7, 8]. Despite this, the pathogenesis behind the condition remains unclear [4, 9].

One theory is that SARS-CoV-2 infection causes an inappropriate immune response that leads to the varied symptoms of long COVID. This arose from evidence of a marked and persistent increase in autoantibodies in patients with COVID-19 compared to uninfected controls and high rates of patients hospitalised with COVID-19 being transiently positive for anti-phospholipid (aPL) antibodies [10, 11].

Some of these autoantibodies were also deemed as potential risk factors for long COVID [12]. Several systematic reviews have collated case reports of patients with a history of COVID-19 who have experienced deranged immune manifestations. Tang et al. found 187 reports and Novelli et al. found 382 reports of autoimmune-like phenomena following COVID-19 [13, 14]. Among those with a history of COVID-19, one review reported thyroid dysfunction in up to 20% of patients, which is linked with B and T-cell autoimmunity [15].

Autoimmunity may be due to the degree of homology existing between some human self-proteins and components of SARS-CoV-2, a phenomenon termed molecular mimicry [16]. Molecular mimicry combined with the immune system dysregulation that occurs during SARS-CoV-2 infection may be the mechanism driving the development of immune-mediated inflammatory diseases. Alternatively, the reaction could arise from tissue damage and the release of autoantigens as a result of SARS-CoV-2 infection.

This preliminary evidence has been derived largely from case series, case reports, small cohort studies, or systematic reviews of these study types, which are weak study designs for ascertaining causal inference. Stronger study designs are needed that include appropriate control groups and large sample sizes. Furthermore, the data were drawn largely from patients with moderate or severe COVID-19, which underrepresents the mild or asymptomatic cases that make up most SARS-CoV-2 infections and that can also go on to develop long COVID [17]. To address these limitations, we conducted a retrospective matched cohort study using data from a large primary care database to assess the incidence of immune-mediated inflammatory diseases (IMIDs) in patients with SARS-CoV-2 infection compared to matched individuals with no record of SARS-CoV-2 infection.

## Methods

### Study design and data source

A retrospective cohort study was undertaken using data extracted from the Clinical Practice Research Datalink (CPRD) Aurum database between the 31st of January 2020 and the 30th of June 2021. The CPRD Aurum database consists of routinely collected, pseudo-anonymised data from general practices across England [18]. The data were extracted using the data extraction for epidemiological research (DExtER) tool, which facilitates extraction based on predefined parameters [19].

### Study population

Patients were eligible to enter the study if they were at least 18 years old at the study start date, had no prior history of the IMIDs included in the primary outcome (see below), had an acceptable patient flag indicating provision of good quality data, and if they were registered with an eligible general practice for at least 12 months to allow sufficient time for recording baseline information.

### Exposure

All patients with a SNOMED-CT coded diagnosis of either a positive reverse transcriptase polymerase chain reaction (RT-PCR) or lateral flow antigen test for SARS-CoV-2 were included in the exposed cohort, and the date of coded diagnosis was assigned as the index date. Patients with a suspected COVID-19 diagnosis were not included to increase the specificity of the exposure definition. For each exposed patient, up to four patients were selected who did not have a coded record of a positive RT-PCR or lateral flow antigen test, or a diagnosis of suspected or confirmed diagnosis of COVID-19, and were matched on age, sex and registered general practice. This made up the unexposed cohort. The same index date of the exposed patients was assigned to the corresponding matched unexposed patients to avoid immortal time bias [20]. Data from the COVID-19 Second Generation Surveillance System was not used for this study as it comprised of data from swab testing in Public Health England (PHE) labs and NHS hospitals primarily for hospitalised patients and healthcare workers as opposed to data from the wider population which was required for this study.

### Outcomes

The primary outcome was a composite of the incidence of any of the following IMIDS: autoimmune thyroiditis, coeliac disease, inflammatory bowel disease (IBD), myasthenia gravis, pernicious anaemia, psoriasis, rheumatoid arthritis (RA), Sjogren’s syndrome, systemic lupus erythematosus (SLE), type 1 diabetes mellitus (T1DM), and vitiligo. These conditions were selected as they cover a range of different systems and constitute many of the most prevalent IMIDs in the UK. The secondary outcomes were the individual diseases included in the primary outcome, to discern which of these IMIDs, if any, had the strongest association with SARS-CoV-2 infection. SNOMED-CT code lists used for the ascertainment of each IMID, as well as the exposure codes, are given at https://github.com/Umer-Syed/COVIDAutoimmune. In light of the CPRD policy on data governance, we have not reported outcomes that had below five events due to disclosure risk.

### Follow-up period

Participants were followed up from the index date to the end of the follow-up. The end of follow-up was defined as the earliest of any of the following: a coded diagnosis of an IMID, date of death, study end date (30 June 2021), date of practice de-registration, and date of the last practice contribution to the CPRD Aurum database.

### Covariates

Age, sex, body mass index (BMI), smoking status, ethnicity, previous exposure to relevant viral infections (Epstein-Barr virus (EBV), human cytomegalovirus (CMV), human herpesvirus 6 (HHV-6), human T lymphotropic virus type 1 (HTLV-1), hepatitis C virus (HCV), influenza A virus, and parvovirus B19), and previous prescriptions of selected medications (procainamide, hydralazine, quinidine, and isoniazid) were included as potential confounders. Previous studies found these variables to be associated with at least one of the outcome IMIDs and were thus adjusted for in the analysis [21–33].

Age was divided into the following bands: 18 to 29, 30 to 39, 40 to 49, 50 to 59, 60 to 69, and ≥ 70 years. Ethnicity was identified through SNOMED CT codes and was classified into the following groups: white, South Asian, black, mixed ethnicity and other. BMI was divided in accordance with the WHO classification: underweight (body mass index (BMI) < 18.5 kg/m^2^), normal weight (18.5–24.9 kg/m^2^), overweight (25–29.9 kg/m^2^) and obese (≥ 30 kg/m^2^) [34]. Smoking status was categorised as current smoker, ex-smoker and never smoked. A separate ‘data missing’ category was used where data were missing for ethnicity, smoking status, and BMI.

### Statistical analysis

Baseline characteristics of patients stratified by their exposure status were summarised using simple descriptive statistics. The number and percentage of each of the outcome events for the unexposed and exposed cohorts were reported and the crude incidence rates per 1000 person-years were calculated. Cox proportional hazards regression models were used to estimate the unadjusted and adjusted hazard ratios (HRs) with 95% confidence intervals (CI), for each of the outcomes among patients in the exposed and unexposed cohorts. *P*-values below 0.05 were considered statistically significant. In order to ensure our analysis was valid, a calculation to determine the Schoenfeld residual was undertaken. If this test yielded a value of < 0.05, then the data was not normally distributed and thus the proportional hazard assumption would not be met. All analyses were conducted using Stata Version 17, the do-file for this is given at https://github.com/Umer-Syed/COVIDAutoimmune.

## Results

### Study population

We identified 458,147 patients with confirmed SARS-CoV-2 infection and matched them to 1,818,929 patients who lacked a confirmed or suspected diagnosis of COVID-19. Table 1 shows the baseline characteristics of patients in both cohorts. The mean age was 43.6 years (SD 17.1) in the exposed cohort and 42.8 (SD 18.0) in the unexposed cohort. Both groups had slightly more females than males (54.7% versus 45.3%, respectively). A slightly larger proportion of the exposed cohort were of white and South Asian ethnicity compared to the unexposed group (64.4% versus 59.4%, and 12.2% versus 10.6%, respectively). However, the unexposed cohort had a slightly higher amount of missing ethnicity data (21.6% versus 16.2%, respectively). The mean BMI was similar between groups but there were slightly more current smokers in the unexposed cohort (26.5% versus 22.1%, respectively). Exposure to the selected infections and medications was similar between both groups.

**Table 1:** Baseline characteristics of the exposed and unexposed cohorts

**Characteristics**	**Exposed cohort (*n* = 458,147)**	**Unexposed cohort (*n* = 1,818,929)**
**Sex, *n* (%)**		
**Male**	207,514 (45.3)	823,988 (45.3)
**Female**	250,633 (54.7)	994,941 (54.7)
**Age (years)**		
**Mean (SD)**	43.6 (17.1)	42.8 (18.0)
**Age categories, *n* (%)**		
**18–29**	113,618 (24.8)	478,811 (26.3)
**30–39**	94,499 (20.6)	377,117 (20.7)
**40–49**	87,707 (19.1)	348,335 (19.2)
**50–59**	84,454 (18.4)	327,230 (18.0)
**60–69**	41.841 (9.1)	153,227 (8.4)
** ≥ 70**	36,028 (7.9)	134,209 (7.4)
**Ethnicity, *n* (%)**		
**White**	295,067 (64.4)	1,079,656 (59.4)
**South Asian**	55,792 (12.2)	192,600 (10.6)
**Black**	19,226 (4.2)	86,040 (4.7)
**Mixed ethnicity**	7066 (1.5)	34,134 (1.9)
**Other**	6614 (1.4)	33,873 (1.9)
**Missing**	74,382 (16.2)	392,626 (21.6)
**BMI**		
**Mean (SD)**	27.6 (6.3)	26.8 (6.1)
**Median (IQR)**	26.7 (23.3–30.9)	25.8 (22.5–29.8)
**BMI categories, *n* (%)**		
**Underweight (< 18.5 kg/m**^**2**^**)**	12,038 (2.6)	59,644 (3.3)
**Normal weight (18.5–25 kg/m**^**2**^**)**	136,480 (29.8)	594,657 (32.7)
**Overweight (25–30 kg/m**^**2**^**)**	131,076 (28.6)	473,031 (26.0)
**Obese (> 30 kg/m**^**2**^**)**	116,836 (25.5)	366,198 (20.1)
**Missing**	61,717 (13.5)	325,379 (17.9)
**Smoking status, *n* (%)**		
**Never smoked**	168,415 (36.8)	657,775 (36.2)
**Ex-smoker**	167,507 (36.6)	548,695 (30.2)
**Current smoker**	101,351 (22.1)	482,322 (26.5)
**Missing**	20,874 (4.6)	130,137 (7.2)
**Exposure to selected infection, *n* (%)**		
**All infections**	2354 (0.5)	9098 (0.5)
**EBV**	153 (0.0)	579 (0.0)
**CMV**	123 (0.0)	327 (0.0)
**HHV-6**	0 (0.0)	4 (0.0)
**HTLV-1**	0 (0.0)	10 (0.0)
**Hepatitis C virus**	784 (0.2)	4161 (0.2)
**Influenza A virus**	968 (0.2)	2911 (0.2)
**Parvovirus B19**	282 (0.1)	947 (0.1)
**Exposure to selected medication, *n* (%)**		
**All medications**	821 (0.2)	2552 (0.1)
**Procainamide**	0 (0.00)	2 (0.0)
**Hydralazine**	218 (0.1)	753 (0.0)
**Quinidine**	26 (0.0)	92 (0.0)
**Isoniazid**	581 (0.1)	1724 (0.1)

### Primary analysis

**Fig. 1:**
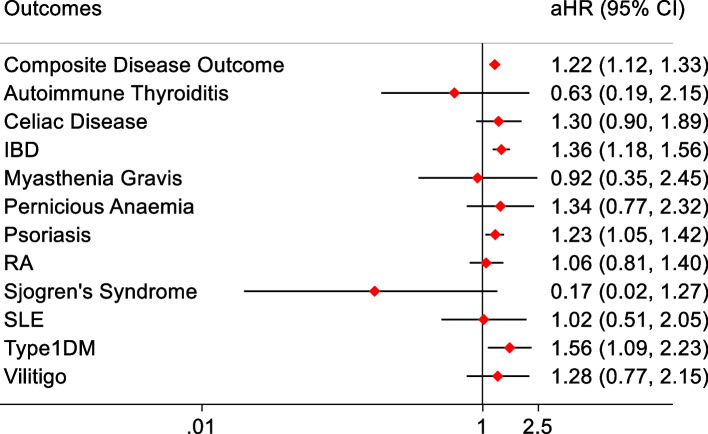
Forest plot of Adjusted HRs for IMIDs. *aHR = Adjusted Hazard Ratio, CI = Confidence Interval, IBD = inflammatory bowel disease, RA = rheumatoid arthritis, SLE = systemic lupus erythematous, Type1DM = type 1 diabetes mellitus

Six hundred ninety-six (0.15%) patients in the exposed cohort developed the primary outcome compared to 2230 (0.12%) within the unexposed cohort. The median (interquartile range [IQR]) follow-up was 0.29 years (0.24–0.42) for both groups. The results of the primary analysis are reported in Table 2 and Fig. 1. The crude incidence rate (IR) per 1000 person-years was higher for the exposed cohort than the unexposed cohort (4.59 versus 3.65 per 1000 person-years, respectively). This yielded a crude hazard ratio of 1.26 (95% CI 1.16–1.37) for the composite primary outcome. When adjusted for pre-selected covariates, the HR slightly reduced to 1.22 (1.12–1.33) but remained statistically significant. The proportional hazard assumption was met based on Schoenfeld residuals for the composite outcome*.* Furthermore, a matched analysis yielded a hazard ratio of 1.25 (95% CI 1.15–1.36). Characteristics of patients stratified by their primary outcome status have been tabulated in Additional file 1: Supplementary Table 2.

**Table 2:** Incidence rates and HRs for the composite outcome

**Outcome**	**Exposed cohort (*n* = 458,147)**	**Unexposed cohort (*n* = 1,818,929)**
**Outcome events, no. (%)**	696 (0.15)	2230 (0.12)
**Person-years of follow-up**	151,569	611,211
**Crude incidence rate per 1000 person-years**	4.59	3.65
**Median follow-up, years (IQR)**	0.29 (0.24–0.42)	0.29 (0.24–0.42)
**Crude HR (95% CI)**	1.26 (1.16–1.37)	
**Adjusted**^**a**^** HR (95% CI)**	1.22 (1.12–1.33)	

*CI* confidence interval, *HR* hazard ratio, *IQR* interquartile range

^a^Adjusted for age, sex, BMI, ethnicity, smoking status, selected viral infections, and selected medications

### Secondary analysis

Table 3 and Fig. 1 report the results for each individual IMID as separate outcomes. Of the eleven conditions, SARS-CoV-2 infection was significantly associated with an increased incidence of T1DM, IBD and psoriasis. T1DM was 56% more likely to occur in the exposed cohort compared to the unexposed cohort (aHR 1.56, 95% CI 1.09 to 2.23). IBD was 36% more likely to occur in the exposed cohort compared to the unexposed cohort (aHR 1.36, 95% CI 1.18 to 1.56). This was the most common IMID to be diagnosed during the study period (39.6% of all IMIDs diagnosed in the exposed cohort and 36.6% in the unexposed cohort). Psoriasis was 23% more likely to occur in the exposed cohort compared to the unexposed cohort (1.23, 1.05 to 1.42) and was the second most diagnosed IMID, representing more than 30% of all new diagnoses of IMIDs in both cohorts.

**Table 3:** Incidence rates and hazard ratios for individual IMIDs

**Outcome**	**Exposed cohort**	**Unexposed cohort**
**Autoimmune thyroiditis**		
Outcome events	*N* < 5	18
Crude incidence rate per 1000 person-years	0.020	0.029
Crude HR (95% CI)	0.67(0.20–2.27)	
Adjusted HR (95% CI)	0.63 (0.19–2.15)	
**Coeliac disease**		
Outcome events	37	113
Crude incidence rate per 1000 person-years	0.24	0.18
Crude HR (95% CI)	1.32 (0.91–1.92)	
Adjusted HR (95% CI)	1.30 (0.90–1.89)	
**Inflammatory bowel disease**		
Outcome events	276	817
Crude incidence rate per 1000 person-years	0.18	0.13
Crude HR (95% CI)	1.37 (1.19–1.57)	
Adjusted HR (95% CI)	1.36 (1.18–1.56)	
**Myasthenia gravis**		
Outcome events	5	21
Crude incidence rate per 1000 person-years	0.033	0.034
Crude HR (95% CI)	0.96 (0.36–2.55)	
Adjusted HR (95% CI)	0.92 (0.35–2.45)	
**Pernicious anaemia**		
Outcome events,	17	50
Crude incidence rate per 1000 person-years	0.11	0.082
Crude HR (95% CI)	1.37 (0.79–2.38)	
Adjusted HR (95% CI)	1.34 (0.77–2.32)	
**Psoriasis**		
Outcome events	223	743
Crude incidence rate per 1000 person-years	1.47	1.22
Crude HR (95% CI)	1.21 (1.04–1.41)	
Adjusted HR (95% CI)	1.23 (1.05–1.42)	
**Rheumatoid arthritis**		
Outcome events	66	248
Crude incidence rate per 1000 person-years	0.44	0.41
Crude HR (95% CI)	1.08 (0.82–1.41)	
Adjusted HR (95% CI)	1.06 (0.81–1.40)	
**Sjogren’s syndrome**		
Outcome events	*N* < 5	23
Crude incidence rate per 1000 person-years	0.00066	0.038
Crude HR (95% CI)	0.18 (0.02–1.31)	
Adjusted HR (95% CI)	0.17 (0.02–1.27)	
**Systemic lupus erythematosus**		
Outcome events, no. (%^a^)	10	39
Crude incidence rate per 1000 person-years	0.066	0.064
Crude HR (95% CI)	1.03 (0.51–2.06)	
Adjusted HR (95% CI)	1.02 (0.51–2.05)	
**Type 1 diabetes mellitus**		
Outcome events	42	106
Crude incidence rate per 1000 person-years	0.28	0.17
Crude HR (95% CI)	1.60 (1.12–2.29)	
Adjusted HR (95% CI)	1.56 (1.09–2.23)	
**Vitiligo**		
Outcome events	19	60
Crude incidence rate per 1000 person-years	0.13	0.098
Crude HR (95% CI)	1.28 (0.76–2.14)	
Adjusted HR (95% CI)	1.28 (0.77–2.15)	

*CI* confidence interval, *HR* hazard ratio

^a^Total outcome events

## Discussion

### Main findings

Exposure to SARS-CoV-2 infection was associated with a 22% relative increase in the incidence of any of the eleven IMIDs considered in our study compared to a matched unexposed group during the same period. This was after adjustment for several important confounding factors and during a relatively short period of follow-up. We also found that this association was specific to an increased incidence of T1DM, inflammatory bowel disease, and psoriasis in the SARS-CoV-2 infected cohort.

### Comparison with existing literature

The relatively high incidence of psoriasis in the SARS-CoV-2 infected cohort is supported by other reports from the literature which found increased cases of psoriasis, and flares of existing disease, following COVID-19 [13]. Evidence on the incidence of IBD following COVID-19 is scarcer, although ulcerative colitis has been reported to develop post-infection [13]. A systematic review on T1DM and COVID-19 noted that between 1.77 and 15.6% of newly diagnosed patients, depending on the study, had preceding COVID-19 [35].

SARS-CoV-2 may be associated with IMIDs due to several putative mechanisms that result in the release of autoantibodies following infection. All three conditions that were found to have a significantly increased incidence following SARS-CoV-2 infection in our study have at least a limited association with autoantibodies. T1DM is associated with islet cells and other autoantibodies, psoriasis is linked with anti-nuclear antibodies (ANAs) and inflammatory bowel disease has a limited association with pancreatic autoantibodies (PAB) [36–38]. The reason for the increased incidence of these conditions following SARS-CoV-2 infection is unclear as they are not typically the most strongly associated with the presence of autoantibodies. This requires further exploration in future mechanistic studies.

### Strengths and limitations

A large sample size was included, which provided sufficient statistical power to assess for differences in the incidence of IMIDs between the exposed and unexposed cohorts over a relatively short follow-up period. This also allowed us to assess the relative incidence of eleven of the more common IMIDs across the two comparison groups. We included IMIDs in our outcome such as T1DM, that are likely to be well-recorded in primary care records. The use of primary care data meant that we were able to adjust for important demographic and clinical risk factors that are known to be associated with the incidence of IMIDs. The use of data from practices across a national database also improved the generalisability of our findings.

The study had several limitations. We had missing data for ethnicity (22% missing), BMI (18%), and smoking status (7%), which we accounted for in our analyses using a missing category variable. However, these missing data could lead to biased effect estimates. We also did not have access to data on socioeconomic status but partially accounted for this by matching patients in the unexposed and exposed cohorts on general practice, which would result in patients from both groups sharing their approximate residential geography, which is associated with socioeconomic status.

There is likely to be a degree of misclassification bias between the exposed and unexposed cohorts. There was little community testing for SARS-CoV-2 infection in the first wave of the pandemic, so some members of the unexposed cohort may have been infected but not diagnosed. IMIDs may also have been underdiagnosed during the study period due to the relative inaccessibility of healthcare services during the early phase of the pandemic. It is possible that only more severely affected patients with IMIDs presented to healthcare services during this period.

The study period was restricted as data availability only covered from 31 January 2020 to 30 June 2021. This encompassed three national lockdowns where reduced healthcare appointments led to a backlog of up to 300,000 patients waiting over a year for treatment [39, 40]. Beyond this period, there was reduced availability of community testing for SARS-CoV-2 infection in the UK at a time when an increasing proportion of the population had experienced at least one episode of COVID-19, thus diminishing future comparator populations.

The short follow-up period may have diluted the effect size and power of the study as IMIDs tend to have a clinical latency period and thus the full scope of the potential impact of SARS-CoV-2 infections is likely to have been underrepresented [41]. It also cannot be confirmed whether the true onset of these conditions preceded SARS-CoV-2 infection or the matched index dates. However, we would expect these issues to equally bias our estimates of disease incidence in both the exposed and unexposed cohorts and would therefore not anticipate it affecting the hazard ratios. There also exists the possibility that patients experiencing COVID-19 may have accessed healthcare services more than those with no prior infection and thus had more opportunities to be diagnosed with IMIDs. Likewise, patients with underlying IMIDs may have had their symptoms exacerbated by COVID-19 which resulted in seeking healthcare services and subsequent diagnosis.

### Implications for practice, policy, and research

Our findings provide epidemiological evidence that SARS-CoV-2 infection is associated with an increased risk of a range of IMIDs, including T1DM, IBD, and psoriasis. This provides evidence that autoimmunity may be a potential mechanism that accounts for some of the longer-term symptoms and health impacts of a subgroup of those with long COVID. This is particularly of interest given the finding that women are generally at increased risk of both IMIDs as well as Long COVID, that symptoms of long COVID are diverse and often overlap with those of IMIDs, and that the symptoms of both IMIDs and long COVID characteristically follow a relapsing–remitting pattern over time [42].

Further epidemiological studies with a longer follow-up period are needed to confirm our findings and to test for relevant autoantibodies in the serum of participants to correlate with symptoms and clinical findings. These studies could also include other rarer IMIDs potentially associated with COVID-19 such as Guillain-Barré syndrome [14]. Evidence suggests that those who have been vaccinated against COVID-19 are approximately half as likely to develop symptoms lasting over 28 days than unvaccinated individuals [43]. It would be valuable to know if these differences in long COVID incidence rates are also associated with differences in the incidence of IMIDs.

## Conclusions

SARS-CoV-2 infection was associated with an increased incidence of several IMIDs, including type 1 diabetes mellitus, inflammatory bowel disease, and psoriasis. This lends support to the hypothesis that the long-term effects of COVID-19 or long COVID may in part be related to autoimmune mechanisms. Further research is needed to replicate these findings in other populations, over a longer time period and to sample autoantibody profiles in people with long COVID and matched control groups.

### Supplementary Information

**Additional file 1: Supplementary Table 1.** Adjusted hazard ratios for other risk factors included in the Cox proportional hazards model. **Supplementary Table 2.** Population characteristics by outcome cohort. The RECORD statement: A checklist of items, extended from the STROBE statement, that should be reported in observational studies using routinely collected health data.

## Data Availability

Access to anonymized patient data from CPRD is subject to a data-sharing agreement containing detailed terms and conditions of use following protocol approval from the MHRA Independent Scientific Advisory Committee. The dataset for this study is not publicly available but may be requested from the corresponding author at a.subramanian@bham.ac.uk if in accordance with data governance approvals. Details about Independent Scientific Advisory Committee applications and data costs are available on the CPRD website (cprd.com).
